# Econsult system: direct coordination between mental health and primary care

**DOI:** 10.1192/j.eurpsy.2025.1429

**Published:** 2025-08-26

**Authors:** A. Izquierdo De La Puente, P. Del Sol Calderon, R. Fernandez Fernandez, T. Gonzalez Salvador, M. García Moreno

**Affiliations:** 1 Psiquiatria, Hospital Universitario Puerta de Hierro de Majadahonda; 2 Psiquiatria, Hospital Universitario Infanta Cristina, Madrid, Spain

## Abstract

**Introduction:**

The econsulta is the telematic coordination system between hospital care and primary care. In this way, it allows an agile and fluid coordination, improving the coordination and the care process of the patient.

**Objectives:**

With the data collected and analyzed, a descriptive study of the functioning of the econsulta as a coordination tool between Mental Health and Primary Care is carried out.

**Methods:**

The data collected corresponds to health area 6 of the Community of Madrid, which consists of 18 primary health centers for a Mental Health Center. The econsultations carried out between January 2022 and August 2024 are analyzed.

The data collected are broken down into patient diagnoses, coded according to the ICD-10 classification system, and the attitude to be taken in each of them.

**Results:**

A total of 1489 econsults performed in the period between January 2022 and August 2024 were obtained. The majority diagnoses are:

- Organic mental disorders, including symptomatic.
- Mental and behavioral disorders due to the use of multiple drugs or other psychotropic substances.
- Schizophrenia, schizotypal disorder and delusional disorders.
- Mood (affective) disorders.
- Mood disorders
- Neurotic disorders, secondary to stressful situations and somatoform disorders.
- Eating behavior disorders.
- Personality and behavioral disorders in adults.
- Mental retardation.
- ADHD

Thanks to the econsult, 692 patients have been resolved, without requiring referral to Mental Health, through direct indications and recommendations on treatments prescribed in Primary Care. Likewise, 61 of them avoided referral only to obtain a visa for the usual treatment. On the other hand, 378 patients required direct referral to Mental Health and 54 to the CAID. In addition, 229 of the patients who were already under follow-up were consulted about their treatment.

After analyzing the data, Primary Care made adequate use of the system, given that only 40 of the consultations were not appropriate.

**Conclusions:**

After analyzing the data, the econsultation is a fast and effective coordination system that increases the efficiency of the patient care system.

**Disclosure of Interest:**

None Declared

